# Clinical features of symptomatic patellofemoral joint osteoarthritis

**DOI:** 10.1186/ar3779

**Published:** 2012-03-14

**Authors:** George Peat, Rachel C Duncan, Laurence RJ Wood, Elaine Thomas, Sara Muller

**Affiliations:** 1 Arthritis Research UK Primary Care Centre, Primary Care Sciences, Keele University, Keele, Staffordshire ST5 5BG, UK

## Abstract

**Introduction:**

Patellofemoral joint osteoarthritis (OA) is common and leads to pain and disability. However, current classification criteria do not distinguish between patellofemoral and tibiofemoral joint OA. The objective of this study was to provide empirical evidence of the clinical features of patellofemoral joint OA (PFJOA) and to explore the potential for making a confident clinical diagnosis in the community setting.

**Methods:**

This was a population-based cross-sectional study of 745 adults aged ≥50 years with knee pain. Information on risk factors and clinical signs and symptoms was gathered by a self-complete questionnaire, and standardised clinical interview and examination. Three radiographic views of the knee were obtained (weight-bearing semi-flexed posteroanterior, supine skyline and lateral) and individuals were classified into four subsets (no radiographic OA, isolated PFJOA, isolated tibiofemoral joint OA, combined patellofemoral/tibiofemoral joint OA) according to two different cut-offs: 'any OA' and 'moderate to severe OA'. A series of binary logistic and multinomial regression functions were performed to compare the clinical features of each subset and their ability in combination to discriminate PFJOA from other subsets.

**Results:**

Distinctive clinical features of moderate to severe isolated PFJOA included a history of dramatic swelling, valgus deformity, markedly reduced quadriceps strength, and pain on patellofemoral joint compression. Mild isolated PFJOA was barely distinguished from no radiographic OA (AUC 0.71, 95% CI 0.66, 0.76) with only difficulty descending stairs and coarse crepitus marginally informative over age, sex and body mass index. Other cardinal signs of knee OA - the presence of effusion, bony enlargement, reduced flexion range of movement, mediolateral instability and varus deformity - were indicators of tibiofemoral joint OA.

**Conclusions:**

Early isolated PFJOA is clinically manifest in symptoms and self-reported functional limitation but has fewer clear clinical signs. More advanced disease is indicated by a small number of simple-to-assess signs and the relative absence of classic signs of knee OA, which are predominantly manifestations of tibiofemoral joint OA. Confident diagnosis of even more advanced PFJOA may be limited in the community setting.

## Introduction

Osteoarthritis (OA) is not a single disease [1] and distinct phenotypes are believed to exist even within a single joint complex like the knee. Among the various approaches to subclassifying knee OA, the recent European League Against Rheumatism (EULAR) Task Force on diagnosis of knee OA recognised that subsets with different risk factors and outcomes can be defined by compartmental distribution, but pointed out that the ability to discriminate between these subsets in routine practice and the utility of doing so had not been formally tested [2]. Perhaps unsurprisingly, the diagnosis of knee OA subsets is rarely seen in current primary care. For example, in a total population of 57,555 adults registered with UK general practices, only 13 cases had a recorded diagnosis by the general practitioner of patellofemoral joint OA; less than 1% of knee consulters in a year [3].

There are several reasons why distinguishing patellofemoral from tibiofemoral joint OA phenotypes may be important. There is growing evidence indicating that patellofemoral joint OA impacts independently on symptoms and function [4-9], that it also frequently occurs in the absence of tibiofemoral disease [4,6,10-13], and that its aetiology and, therefore, risk profile and management, may differ [12,14-17]. For example, a history of knee injury or meniscectomy may tend to indicate tibiofemoral joint OA [14,15] while a history of anterior knee pain in young adulthood may suggest patellofemoral joint OA [18]. The direction of frontal plane knee malalignment may serve to indicate patellofemoral joint OA and tibiofemoral joint OA (valgus malalignment being associated with the predominant pattern of lateral patellofemoral joint OA, varus malalignment with medial tibiofemoral joint OA) [16,19-21]. Although a recent systematic review revealed only two randomised controlled trials of interventions specifically for isolated patellofemoral joint OA [22], more are now emerging [23,24]. In the context of recommendations that OA can often be confidently diagnosed without the need for imaging [2,25], these developments pose a fundamental question: can patellofemoral joint OA be identified in routine clinical practice and, if so, which features are most informative?

Expert clinical accounts of the clinical manifestations and typical features of patellofemoral OA are available in medical textbooks and review articles [17,26-31] but there has been very little empirical research. One exception, a hospital-based case-control study, documented the comparative clinical features of patellofemoral and tibiofemoral joint OA in only 42 knees [32]. Previous research on the clinical features, classification criteria and diagnosis of knee OA, including that for the American College of Rheumatology (ACR) classification criteria and EULAR Task Force, has tended to be based on knee OA as a whole [33-38] and there is some evidence that the features derived from these studies may selectively reflect tibiofemoral disease [39]. A recent editorial concluded that "little is known about how best to separate patellofemoral symptoms from those arising from the tibiofemoral joint" [40].

In this paper we set out to extend our previous work [37] on pursuing rational clinical diagnosis of knee OA in primary care. We investigate the comparative clinical features of symptomatic patellofemoral and tibiofemoral joint OA and we explore their ability, when used in combination, to allow confident diagnosis of subsets of symptomatic knee OA in the community setting.

## Materials and methods

### Study design

This was a cross-sectional diagnostic study in adults aged 50 years and over reporting current or recent knee pain not attributed to inflammatory arthropathy. The reference standard was patellofemoral and/or tibiofemoral joint OA defined using plain radiography. Diagnostic indicators were previously-documented risk factors, and clinical signs and symptoms obtained from a simple, low-cost, non-instrumented assessment.

### Study population

Participants were recruited from a two-stage cross-sectional postal survey of all adults ages ≥50 years registered with three general practices in North Staffordshire (irrespective of actual consulting patterns). Respondents reporting pain of any duration in or around the knee within the previous 12 months were invited to attend a research clinic at a local National Health Service Hospital Trust. The study protocol was approved by North Staffordshire Local Research Ethics Committee (project number 1430) and details have been published elsewhere [41,42]. All participants provided written informed consent to undergo clinical and radiographic assessment. In addition, they were asked for consent to medical record review to assist in excluding preexisting inflammatory disease. The inclusion criteria for the current analysis were as follows: age ≥50 years, registered with one of the participating general practices at the time of study, responded to both postal questionnaires, consented to further contact, and attended the research clinic. Participants were excluded if they had not experienced knee pain within the six months prior to clinic attendance, had a pre-existing diagnosis of inflammatory arthropathy in their medical records, or had had a total knee replacement in their most affected knee.

### Data collection

All data were planned and gathered prospectively. Participants underwent a standardized clinical interview and physical examination conducted by one of six research therapists blinded to the findings from radiography, postal questionnaires and medical records. The assessments were abbreviated versions of those developed in an earlier stage of this research through consultation and formal consensus exercises with practising clinicians [43,44]. Inter- and intra-rater reliability and quality assurance and control procedures have been reported elsewhere [37,45,46].

Participants filled in a brief self-complete questionnaire about their knee symptoms on the day of their clinic attendance. Copies of self-complete questionnaires and detailed protocols for clinical assessment are available on request from the corresponding author.

Plain knee radiographs were obtained on the day of clinic attendance. Three views were taken of each knee: a weight-bearing semi-flexed posteroanterior (PA) view, according to the protocol developed by Buckland-Wright *et al. *[47], and lateral and skyline views, both in a supine position with the knee flexed to 45°. The tibiofemoral joint was assessed using the PA view and the posterior compartment of the lateral view. The patellofemoral joint was assessed using the skyline and lateral views.

### Scoring of plain radiographs

A single reader (RD), blinded to all other information on participants, scored all films. Films were scored for individual radiographic features, including osteophytes, joint space width, sclerosis, subluxation and chondrocalcinosis. The atlas and scoring system developed by Altman *et al. *[48,49] were used for the PA and skyline views and the atlas developed by Burnett *et al. *[50] was used for the lateral view. Additionally, PA and skyline views were assigned a Kellgren and Lawrence (K&L) grade based on these authors' original written descriptions [51]. For PA, K&L score, skyline K&L score and lateral osteophytes, intra- and inter-reader reliability were assessed in a subsample of 50 participants (100 knees) and found to be very good (κ = 0.81 to 0.98 and 0.49 to 0.76, respectively) [13].

### Statistical analysis

#### Defining radiographic patellofemoral joint OA and tibiofemoral joint OA

Only one knee per individual was analysed, the "index knee": the single painful knee in participants with unilateral knee pain and the most painful knee in individuals with bilateral knee pain. An individual was allocated to one of four mutually exclusive groups: (1) no radiographic OA, (2) isolated patellofemoral joint OA, (3) isolated tibiofemoral joint OA or (4) combined patellofemoral/tibiofemoral joint OA. We repeated all analyses using two cut-offs for defining the outcome of radiographic OA using the more stringent of the two to attempt to identify 'purer' phenotypes. The operational definitions are provided in Table 1.

**Table 1:** Definitions of subsets of symptomatic radiographic knee OA

	'Any OA'	'Moderate to severe OA'
PFJOA	Skyline K&L ≥2	Skyline K&L ≥3
	OR	OR
	Lateral osteophytes ≥1	Lateral osteophytes = 3

TFJOA	PA K&L ≥2	PA K&L ≥3
	OR	OR
	Posterior osteophytes ≥1	Posterior osteophytes = 3

K&L Kellgren and Lawrence score; OA Osteoarthritis; PA Posteroanterior; PFJ Patellofemoral joint; TFJ Tibiofemoral joint

#### Potential indicators of patellofemoral joint OA and tibiofemoral joint OA

Prior to analysis, a total of 40 potential indicators were identified from information in the two postal questionnaires, the clinical assessment and the brief self-complete questionnaire (Table 2). Potential indicators were chosen, if they were known or suspected risk indicators, for radiographic OA, clinical signs and symptoms with a known or putative link to the occurrence of radiographic OA, or clinical manifestations of alternative diagnoses [37]. All indicators had to be practicable for assessment within a routine primary care consultation. Because of collinearity between items in the Western Ontario and McMaster Universities Osteoarthritis Index (WOMAC) we selected only the first items from the Pain, Stiffness and Function subscales (pain walking on flat surfaces, stiffness on waking, difficulty descending stairs).

**Table 2:** Potential indicators of patellofemoral and tibiofemoral joint OA considered in the current study

Domain	Indicator	Source
***Risk factors***	Age; sex	
	
	1^st ^degree relative with arthritis; previous menisectomy; contralateral TKR	In-clinic face-to-face interview
	
	Body mass index	In-clinic measurement
	
	Clinical hand OA	In-clinic self-complete questionnaire + physical examination

***Clinical history/symptoms***	Time since problem onset; gradual onset; problem started following injury; bilateral knee pain; incident pain; duration of morning stiffness; inactivity gelling; reported swelling in past month; reported dramatic swelling ever; locking; giving way	In-clinic face-to-face interview
	
	Pain days in last six months‡; pain, stiffness, aching on most days in the past month; current pain intensity (0 to 10 NRS)‡; significant interference with daily activities in last six months‡;	In-clinic self-complete questionnaire
	
	Whole leg pain; pain walking on a flat surface†; stiffness on waking†; difficulty descending stairs†	Prior postal self-complete questionnaire¶

***Physical signs***	Intercondylar gap in standing; intermalleolar gap in standing; PFJ glide/compression test; presence and severity of palpable knee effusion; fixed flexion deformity; bony enlargement; mediolateral instability; isometric knee extensor strength; isometric knee flexor strength; coarse crepitus; knee flexion ROM; TF joint line tenderness; multiple local tender points§; timed single-leg standing balance	In-clinic physical examination

‡ Items from Chronic Pain Grade; † Items from Western Ontario and McMaster Universities Osteoarthritis Index (WOMAC) LK3.0, dichotomised at moderate or worse; § four or more from medial femoral condyle, medial TF joint line, lateral femoral condyle, lateral TF joint line, prepatellar, infrapatellar, pes anserinus Completed a median of 53 days prior to research assessment clinic attendance

NRS, numerical rating scale; OA, osteoarthritis; PFJ, patellofemoral joint; ROM, range of movement; TF, tibiofemoral; TKR, total knee replacement

To explore the comparative clinical features of isolated patellofemoral joint OA, isolated tibiofemoral joint OA and combined patellofemoral/tibiofemoral joint OA, a series of pairwise binary logistic regression models were undertaken based on complete case analyses (missing data on indicators was < 1% except for WOMAC items (< 7%)). The strength of association between each potential indicator and outcome was initially evaluated adjusting for age, gender and measured body mass index. Variables with a *P*-value < 0.05 for the likelihood ratio test were considered eligible for entry into a multivariable model. Where different items addressed the same underlying clinical construct (for example, patient-perceived swelling) one variable was selected to represent that construct and was entered into the multivariable model. Binary logistic regression was used to fit the multivariable model, with age, gender and body mass index forced into the model, and a backward elimination procedure (*P *= 0.05) used for variable reduction. The final models were refitted to participants with complete data on the retained predictor variables. Model calibration was checked using the Hosmer-Lemeshow goodness of fit statistic. Model discrimination was summarised by the area under the receiver operator characteristic (ROC) curve (AUC) and was visually displayed using simple histograms of density functions, which show the distribution and overlap in predicted probabilities generated from the logistic regression models [52].

Finally, to explore the ability of these clinical variables to support a confident diagnosis of patellofemoral and tibiofemoral joint OA we fitted a multinomial regression function, with the isolated patellofemoral group as the reference, using the indicators identified from the above pairwise analyses. For this purpose, categorical indicators were dichotomised. Once again, age, gender and body mass index were forced into the model, and a backward elimination procedure (*P *= 0.05) was used for variable selection. From the predicted probabilities, we summarised the proportion of participants correctly classified on the 'balance of probabilities' (that is, the category with the highest predicted probability) and the numbers of instances where the predicted probability exceeded the arbitrarily chosen threshold of 80% for a confident diagnosis.

Analyses were undertaken in Stata 11.0 (StataCorp, 2009, College Station, Texas, USA) and PASW 18.0 (PSS Inc., 2010, Chicago, Illinois, USA).

## Results

### Study participants

Between August 2002 and September 2003, 819 people attended the research clinic, of whom 745 were eligible for the current analysis (mean (SD) age 65.2 (8.6) years; 55% female; mean (SD) body mass index 29.6 (5.2) kg/m^2^). Reasons for ineligibility were: participants declining radiography (*n *= 2), incomplete radiographic data (total knee replacement in index knee (*n *= 15), unlabelled PA view (*n *= 2), absent patella (*n *= 2), uninterpretable skyline view (*n *= 5)), existing diagnosis of inflammatory arthritis verified by medical record review (*n *= 16), no knee pain in the last six months (*n *= 32).

### Comparative clinical features: 'any OA'

When applying the lower threshold definition of radiographic OA, the numbers of participants classed as no radiographic OA, isolated patellofemoral joint OA, isolated tibiofemoral OA, and combined patellofemoral/tibiofemoral joint OA were 236 (32%), 178 (24%), 30 (4%) and 301 (40%), respectively. Due to the small number with isolated tibiofemoral joint OA, modelling was limited to comparing the clinical features of no radiographic OA, isolated patellofemoral joint OA and combined patellofemoral/tibiofemoral joint OA.

In addition to age, gender and body mass index, a total of 21 risk factors, clinical signs and symptoms were significantly different on at least one pair-wise comparison (Additional file 1), suggesting them as diagnosis-relevant indicators. Due to small numbers we were unable to include previous menisectomy or total knee replacement in the contralateral knee.

Additional file 2 shows the results from the multivariable regression functions for each pair-wise comparison.

#### Isolated patellofemoral joint OA vs No radiographic OA

The regression function for isolated patellofemoral joint OA compared to no radiographic OA had the lowest AUC and greatest overlap in predicted probabilities. Difficulty descending stairs (adjusted OR 1.83; 95% CI 1.13, 2.96) and the presence of coarse crepitus (definite crepitus: aOR 2.46; 1.32, 4.60) were marginally informative when added to age, sex and body mass index but added little discriminative power (AUC 0.71 (95% CI 0.66, 0.76) vs 0.69 (0.64, 0.74); Χ^2 ^= 1.23; *P *= 0.264).

#### Combined patellofemoral/tibiofemoral joint OA vs no radiographic OA

Combined patellofemoral/tibiofemoral joint OA was distinguished from no radiographic OA by older age, higher body mass index, patient-reported onset following injury (aOR 2.18; 1.07, 4.44), stiffness on waking (1.92; 1.10, 3.34), difficulty descending stairs (2.53; 1.40, 4.57), palpable effusion (for example, mild effusion: 3.08; 1.75, 5.42), fixed flexion deformity (7.58; 2.08, 27.58), coarse crepitus (for example, definite crepitus: 3.38; 1.75, 6.55) and lower knee flexion range of motion (0.96; 0.94, 0.99). Female gender and the patient-reported whole leg pain (0.28; 0.13, 0.61) tended to indicate no radiographic OA.

#### Combined patellofemoral/tibiofemoral joint OA *vs *Isolated patellofemoral joint OA

Compared with isolated patellofemoral joint OA, individuals with combined patellofemoral/tibiofemoral joint OA were more likely to be older, female, obese and have varus deformity (2.11; 1.18, 3.75), palpable effusion (for example, mild effusion: 2.82; 1.70, 4.69), bony enlargement (for example, definite bony enlargement: 3.01; 1.56, 5.81), fixed flexion deformity (2.11; 1.04, 4.28) and lower knee flexion range of motion on examination (0.96; 0.94, 0.99).

In the final multinomial model, with the isolated patellofemoral group as the reference, the probability of subtypes of any knee OA was a joint function of age, **gender**, body mass index, patient-reported whole leg pain and difficulty descending stairs and, on physical examination, intercondylar gap, palpable effusion, fixed flexion deformity, bony enlargement, coarse crepitus and knee flexion range of motion (Table 3). Classification based on the 'balance of probabilities' was correct in 392 (67%) instances. A confident (≥80% probability) correct diagnosis of isolated patellofemoral joint OA and combined patellofemoral/tibiofemoral joint OA was possible in 0 and 79 (28%) cases, respectively.

**Table 3:** Multinomial regression function: 'any OA'

N	666		
Missing	49		
	**NONE**	**ISO-PF**	**COMB**
	***n *= 212**	***n *= 170**	***n *= 284**
	aOR (95% CI)*		aOR (95% CI)*

Age (per year)	0.96 (0.93, 0.99)	1	1.04 (1.01, 1.07)
Female gender	2.87 (1.79, 4.59)	1	1.56 (0.99, 2.47)
BMI (per kg/m^2^)	0.94 (0.89, 0.99)	1	1.04 (0.99, 1.09)
Whole leg pain	2.12 (1.10, 4.08)	1	0.74 (0.37, 1.50)
Difficulty descending stairs	0.45 (0.27, 0.73)	1	1.15 (0.74, 1.80)
Intercondylar gap > 0 cm	1.38 (0.75, 2.57)	1	2.34 (1.32, 4.14)
Knee effusion†	0.82 (0.49, 1.35)	1	2.42 (1.55, 3.77)
Fixed flexion deformity	0.20 (0.04, 0.93)	1	2.31 (1.13, 4.77)
Coarse crepitus‡	0.64 (0.40, 1.02)	1	1.57 (1.03, 2.41)
Knee flexion ROM (per degree)	0.99 (0.96, 1.01)	1	0.96 (0.94, 0.98)

Nagelkerke's Pseudo R^2^	0.41		
Pearson goodness-of-fit	*P *= 0.45		
Pre-test probability	0.32	0.26	0.43

Correctly classified on 'balance of probabilities'	136 (64%)	36 (21%)	220 (77%)
Correctly classified on 'confident diagnosis' (probability ≥0.8)	18 (8%)	0	79 (28%)

* odds ratio with 95% confidence interval, adjusted for all other covariates in model. aOR is interpreted as the odds relative to the ISO-PF group (for example, compared to those people with ISO-PF, people with COMB have 2.4 times the odds of having knee effusion; compared with ISO-PF, those with no ROA have 0.45 times the odds of difficulty descending stairs, that is, lower risk).

†Mild/Moderate/Gross

‡Possible/definite

### Comparative clinical features: 'moderate to severe OA'

When applying the more stringent cut-off for radiographic OA ('moderate to severe OA'), the numbers of participants classed as no/mild radiographic OA, isolated patellofemoral joint OA, isolated tibiofemoral joint OA and combined patellofemoral/tibiofemoral joint OA were 453 (61%), 99 (13%), 123 (17%) and 70 (9%), respectively.

In addition to age, gender and body mass index, a total of 26 risk factors, clinical signs and symptoms were significantly different on at least one pair-wise comparison (Additional file 3), suggesting them as diagnosis-relevant indicators. This list of 26 potential indicators included all but two (patient-reported whole leg pain and incident pain) of those found to be associated in the 'any OA' models and seven additional indicators (patient-reported locking and significant interference with activities and, on physical examination, intermalleolar gap, pain on patellofemoral joint glide/compression, quadriceps strength, multiple local tender points, and timed single-leg standing balance).

Additional file 4 shows the multivariable regression functions for each pair-wise comparison.

All groups with moderate to severe knee OA were older and more obese than those with no-mild radiographic osteoarthritis (ROA). However, neither age nor body mass index appeared to differ between subsets with 'moderate to severe OA' after adjustment for covariates. Patient-perceived onset following injury, intercondylar gap > 0 cm (a crude measure of varus malalignment), palpable effusion, bony enlargement, fixed flexion deformity and lower knee flexion range of motion tended to be associated with tibiofemoral disease. By contrast, a recalled episode of dramatic swelling in the past, intermalleolar gap > 0 cm (valgus malalignment), markedly reduced knee extensor strength, and pain on PFJ compression appeared to indicate patellofemoral joint disease.

In the final multinomial model, with the isolated patellofemoral group as the reference, the probability of subsets of 'moderate to severe OA' was a joint function of age, sex, body mass index, patient-perceived time since the onset and onset following injury, patient-recalled dramatic swelling, self-reported difficulty descending stairs and, on physical examination, varus malalignment, valgus malalignment, pain on patellofemoral joint glide/compression, palpable effusion, fixed flexion deformity, bony enlargement, mediolateral instability, coarse crepitus, quadriceps strength and knee flexion range of motion (Table 4). Classification based on balance of probabilities was correct in 467 (68%) instances. Correct confident diagnosis of isolated patellofemoral joint OA, isolated tibiofemoral joint OA and combined patellofemoral/tibiofemoral joint OA was not possible in any cases.

**Table 4:** Multinomial diagnostic regression function: 'moderate to severe OA'

N	688			
Missing	57			
	**NONE/MILD**	**ISO-PF**	**ISO-TF**	**COMB**
	***n *= 415**	***n *= 91**	***n *= 114**	***n *= 68**
	aOR (95% CI)*		aOR (95% CI)*	aOR (95% CI)*

Age (per year)	0.91 (0.88, 0.95)	1	0.99 (0.95, 1.03)	1.00 (0.96, 1.05)
Female gender	1.20 (0.66, 2.18)	1	1.18 (0.58, 2.39)	1.09 (0.49, 2.44)
BMI (per kg/m^2^)	0.94 (0.87, 1.00)	1	1.02 (0.95, 1.09)	1.04 (0.97, 1.12)
Dramatic swelling ever	0.36 (0.19, 0.69)	1	0.35 (0.16, 0.76)	0.95 (0.44, 2.07)
Difficulty descending stairs	0.60 (0.35, 1.05)	1	1.36 (0.70, 2.66)	1.41 (0.65, 3.07)
Intercondylar gap > 0 cm	1.53 (0.67, 3.51)	1	4.50 (1.87, 10.84)	1.62 (0.56, 4.68)
PFJ compression test†	0.52 (0.29, 0.92)	1	0.40 (0.20, 0.79)	0.22 (0.10, 0.48)
Knee effusion‡	0.42 (0.25, 0.72)	1	1.21 (0.66, 2.25)	1.29 (0.63, 2.63)
Fixed flexion deformity	0.83 (0.31, 2.24)	1	2.68 (1.01, 7.08)	4.56 (1.67, 12.49)
Bony enlargement§	1.13 (0.66, 1.95)	1	1.75 (0.92, 3.32)	2.59 (1.21, 5.53)
Reduced knee extensor strength¶	0.38 (0.20, 0.69)	1	0.41 (0.20, 0.83)	0.68 (0.30, 1.56)
Coarse crepitus§	0.37 (0.22, 0.63)	1	0.75 (0.40, 1.38)	0.82 (0.40, 1.69)
Knee flexion ROM (per degree)	0.99 (0.96, 1.02)	1	0.96 (0.93, 0.99)	0.95 (0.92, 0.98)

Nagelkerke's Pseudo R^2^	0.47			
Pearson goodness-of-fit	*P *= 1.00			
Pre-test probability	0.60	0.14	0.16	0.10

Correctly classified on 'confident diagnosis' (probability ≥0.8)	222 (53%)	0	0	0
Correctly classified on 'balance of probabilities'	385 (93%)	23 (25%)	41 (36%)	18 (26%)

* odds ratio with 95% confidence interval, adjusted for all other covariates in model. aOR is interpreted as the odds relative to the ISO-PF group (for example, compared with those people with ISO-PF, those with ISO-TF have 4.5 times the odds of intercondylar gap > 0 cm (varus deformity); compared with ISO-PF participants with NONE/MILD, ISO-TF, and COMB were less likely to report pain on patellofemoral joint compression). †Compression or glide pain. ‡Mild/Moderate/Gross. §Possible/Definite. ¶< 141mmHg

## Discussion

While there is little to distinguish mild isolated patellofemoral joint OA from simple knee pain, moderate to severe isolated patellofemoral joint OA is indicated by a history of dramatic swelling in the past, valgus malalignment, markedly reduced quadriceps strength and pain on patellofemoral joint compression. Tibiofemoral joint involvement is indicated by previous injury, varus malalignment, bony enlargement, reduced knee flexion range of motion and fixed flexion deformity. However, in the community setting, confident clinical diagnosis of any subset of radiographic knee OA will often not be possible.

Using comprehensive plain radiographic views - the reference standard recommended by the EULAR Task Force [2] - the current study applied two different thresholds for defining knee OA subsets. We considered a wide range of potential indicators derived from a review of previous literature and consensus development with clinicians and gathered by trained assessors using simple, practicable techniques according to standardised protocols.

Our findings on the pattern of associations between individual risk factors and different subsets of knee OA are largely consistent with those identified in previous longitudinal studies on patellomoral and tibiofemoral joint OA. Age and BMI are confirmed as strong indicators of knee OA but, as McAlindon *et al. *[15] observed, are equally important to patellofemoral and tibiofemoral joint disease subsets. Malalignment is a strong indicator of moderate to severe knee OA subsets with varus malalignment indicating isolated tibiofemoral joint OA and valgus malalignment indicating isolated patellofemoral joint OA. Due to limited numbers of participants, we did not separately define medial and lateral compartment disease for either tibiofemoral or patellofemoral joint OA. Among participants with moderate to severe isolated patellofemoral joint OA in the current study, the ratio of lateral to medial compartment involvement was greater than 2:1. This tendency towards lateral patellofemoral joint disease and its association with valgus malalignment is consistent with previous work [16,19-21,32]. In those with isolated tibiofemoral joint OA, the ratio of medial to lateral compartment disease was greater than 4:1 based on joint space narrowing from the PA view. Given this predominance of medial tibiofemoral joint disease, the association with varus malalignment is consistent with the role of malalignment in the progression of tibiofemoral joint OA [53]. When we narrowed the definition of isolated patellofemoral joint OA to lateral compartment disease only and isolated tibiofemoral joint OA to medial compartment disease only (based on grade 2 to 3 joint space narrowing) the relationship with malalignment became even stronger (see Additional file 5). What our study adds is that the association between malalignment and subsets of knee OA is still detectable even by crude clinical assessment (a gap between the knees or ankles when instructed to stand with the feet together).

Our findings regarding the clinical manifestations of patellofemoral and tibiofemoral joint OA confirm many of those found in previous studies of undifferentiated knee OA. Functional limitation, bony enlargement, coarse crepitus, fixed flexion deformity and reduced flexion range of motion appear to be relatively robust indicators of knee OA [2]. Palpable effusion showed a strong and consistent association with OA in our study, particularly for tibiofemoral joint OA; a finding that contrasts with the EULAR Task Force summary based on two studies [32,34] but which is in agreement with a recent Canadian study [38]. In addition, we found a history of previous dramatic swelling ("came up like a balloon"), markedly reduced quadriceps strength and pain on patellofemoral joint compression were informative indicators of moderate to severe isolated patellofemoral joint OA. Our finding that quadriceps weakness is selectively a feature of more advanced patellofemoral joint OA and not tibiofemoral joint OA appears to support similar recent findings in cross-sectional [54,55] and longitudinal analyses [56] although the markedly reduced performance on isometric testing found as the distinctive feature in the current study may be indicative of painful/fearful inhibition more than weakness *per se*. The finding of a substantially increased risk of dramatic swelling was unanticipated. Without further information we can only speculate on whether this is related to the sorts of previous episodes of subluxation/dislocation reported in hospital cases by Iwano [32].

Our study has several limitations. Plain radiography captures a relatively limited and late view of OA pathology [1]. For that reason, there is the potential for misclassification (specifically due to pre-radiographic tibiofemoral disease) and for the misattribution of clinical features to isolated patellofemoral joint OA [38]. It remains possible, for example, that a recent recalled episode of dramatic swelling, quadriceps weakness or inhibition and reduced knee flexion may be signs not of isolated patellofemoral joint OA but of early tibiofemoral joint OA. While a more sensitive imaging modality would be able to detect this, there is still a fundamental issue that cross-sectional diagnostic studies provide only a snapshot on current status, and in knee OA this is in the context of an evolving sequence (or multiple sequences) of disease [57]. In spite of quality assurance and control procedures, the reliability of the assessment of some clinical signs and symptoms was still poor and would be expected to result in an underestimation of their informativeness. Nevertheless, we feel that this provides a reasonable reflection of what might be expected from non-specialist assessment. Whilst extensive, our list of potential diagnostic indicators was not comprehensive - tenderness on palpation of the patella facets [17] and abnormal gait [38] are two clinical features in particular that might be worth including in future investigations. We initially attempted to image the PF joint in a weight-bearing position according to the Buckland-Wright protocol but this resulted in poor quality films which failed to demonstrate the joint space well in a significant minority of participants who had difficulty adopting the weight-bearing position. Our imaging of the PF joint in the supine position excludes the impact of muscle forces on the joint space width, which may miss minor joint space narrowing. However, this would not affect the classification of 'any' PF OA which relies on the presence of osteophytes. Furthermore, our analysis of "moderate/severe OA" required moderate or worse narrowing and we feel that this is unlikely to have been missed even when the PF joint was imaged in the supine position. Finally, with regard to our multivariable analyses, it should be pointed out that these were based on a high number of variables to cases and with considerable univariable analysis and variable reduction. They require external validation in separate samples. Nonetheless, they do suggest marginally informative clinical features that can be used as the basic building blocks for clinical diagnosis. Our analyses also highlight the oft-neglected issue that even given several 'statistically significant' associations and 'substantial' areas under the ROC curve, one cannot assume that this will translate into the correct classification of a very high proportion of patients or into a confident diagnosis in the majority of cases [58,59]. The best that may be currently achieved by the generalist in routine practice in the absence of definitive imaging is a knowledge of the likely pattern of knee OA based on a 'balance of probabilities'.

## Conclusions

In the case of moderate to severe disease, the clinical profile of symptomatic, radiographically-confirmed patellofemoral joint OA is distinct from tibiofemoral joint OA. However, in the community setting, a confident diagnosis will seldom be possible without imaging. Most signs and symptoms of knee OA reported in the medical literature are predominantly indicators of tibiofemoral joint OA. Selectively effective non-surgical treatments for patello-femoral joint OA are unlikely to be able to be appropriately targeted on clinical grounds alone to the majority of patients presenting with isolated patellofemoral joint OA to primary care.

## Abbreviations

95% CI: 95 percent confidence interval; ACR: American College of Rheumatology; aOR: adjusted odds ratio; AUC: area under the curve; BMI: body mass index; CAS(K): Clinical Assessment Study of the Knee; COMB: combined patellofemoral and tibiofemoral joint OA; EULAR: European League Against Rheumatism; GOF: Goodness of Fit; ISO-PF: isolated patellofemoral joint OA; ISO-TF: isolated tibiofemoral joint OA; K&L: Kellgren and Lawrence; OA: osteoarthritis; NRS: Numerical Rating Scale; PA: posteroanaterior; PFJ: patellofemoral joint; ROA: radiographic osteoarthritis; ROC: receiver operator characteristic; ROM: range of motion; SD: standard deviation; TF: tibiofemoral; TFJ: tibiofemoral joint; TKR: total knee replacement; WOMAC: Western Ontario and McMaster Universities Osteoarthritis Index

## Competing interests

The authors declare that they have no competing interests.

## Authors' contributions

All authors made substantial contributions to the conception and design of the study. GP, RD, ET and LRJW contributed to the acquisition of data. GP, RD and SM performed the statistical analysis. All authors participated in the drafting of the manuscript and read and approved the final manuscript.

## Supplementary Material

Additional file 1 **Descriptive characteristics and univariable analysis: 'any OA'**. Full descriptive characteristics and pairwise univariable comparison of participants with no radiographic OA, isolated patellofemoral joint OA, isolated tibiofemoral joint OA, or combined patellofemoral/tibiofemoral joint OA, using the less stringent cut-off of 'any OA'. Click here for file

Additional file 2 **Content and performance of multivariable models: 'any OA'**. Full reporting, including posterior probability distributions, of the multivariable binary logistic regression models for each pairwise comparison of participants with no radiographic OA, isolated patellofemoral joint OA, or combined patellofemoral/tibiofemoral joint OA, using the less stringent cut-off of 'any OA'. Click here for file

Additional file 3 **Descriptive characteristics and univariable analysis: 'moderate to severe OA'**. This file provides the full descriptive characteristics and pairwise univariable comparison of participants with (1) no radiographic OA, (2) isolated patellofemoral joint OA, isolated tibiofemoral joint OA, or combined patellofemoral/tibiofemoral joint OA, using the more stringent cut-off of 'moderate to severe OA'. Click here for file

Additional file 4 **Content and performance of multivariable models: 'moderate to severe OA'**. Full reporting, including posterior probability distributions, of the multivariable binary logistic regression models for each pairwise comparison of participants with no radiographic OA, isolated patellofemoral joint OA, isolated tibiofemoral joint OA, or combined patellofemoral/tibiofemoral joint OA, using the more stringent cut-off of 'moderate to severe OA'. Click here for file

Additional file 5 **Sensitivity analysis of the association between varus/valgus malalignment and pattern of joint involvement: 'moderate to severe OA'**. Demonstration of the strengthening of association between malalignment and pattern of joint involvement when using compartment-specific definitions. Click here for file
