# Supplementary material for: Clinical features of symptomatic patellofemoral joint osteoarthritis
Source: Arthritis Res Ther. 2012 Mar 14;14(2):R63. doi: 10.1186/ar3779 (PMC3446431; doi:10.1186/ar3779)
Supplement: Additional file 1 — Descriptive characteristics and univariable analysis: 'any OA'. Full descriptive characteristics and pairwise univariable comparison of participants with no radiographic OA, isolated patellofemoral joint OA, isolated tibiofemoral joint OA, or combined patellofemoral/tibiofemoral joint OA, using the less stringent cut-off of 'any OA'. [file ar3779-S1.PDF]

Additional File 1. Descriptive characteristics and univariable analysis: 'any OA'

	Pattern of knee OA				Binary logistic regression†		
	NONE	ISO-PF	ISO-TF	COMB	ISO-PF vs NONE	COMB vs NONE	COMB vs ISO-PF
	n=236	n=178	n=30	n=301	*p*	*p*	*p*
Age, years: mean (SD)	62.1 (7.7)	64.5 (8.0)	63.8 (8.2)	68.3 (8.6)	<0.001	<0.001	<0.001
Female gender	157 (67)	80 (45)	20 (67)	150 (50)	<0.001	0.001	0.182
BMI, kg/m^2 : mean (SD)	28.3 (4.9)	29.4 (4.5)	30.0 (3.3)	30.8 (5.8)	0.003	<0.001	<0.001
1st degree relative with arthritis	152 (64)	110 (62)	23 (77)	169 (56)	0.996	0.679	0.396
Clinical hand OA	43 (18)	32 (18)	5 (17)	52 (17)	0.945	0.554	0.223
Previous menisectomy	2 (1)	4 (2)	0 (0)	20 (7)	-	-	-
Contralateral TKR	2 (1)	1 (1)	0 (0)	9 (3)	-	-	-
Time since onset							
1-5 yrs	85 (36)	67 (38)	11 (37)	94 (31)			
5-10 yrs	50 (21)	35 (20)	4 (13)	61 (20)			
>10 yrs	56 (24)	56 (32)	13 (43)	122 (41)	0.358	<0.001	0.059
Gradual onset	160 (68)	125 (70)	20 (67)	212 (70)	0.298	0.947	0.364
Onset following injury	24 (10)	23 (13)	7 (23)	53 (18)	0.453	0.023	0.056
Whole leg pain	36 (16)	19 (11)	3 (10)	28 (9)	0.124	0.010	0.356
Pain days in past 6mo							
31-89	67 (28)	39 (22)	4 (13)	82 (27)			
90+	43 (18)	47 (26)	4 (13)	107 (36)	0.283	0.003	0.105
Frequent symptoms‡	88 (37)	77 (43)	7 (23)	168 (56)	0.417	0.003	0.062
Bilateral knee pain	169 (72)	143 (80)	19 (63)	230 (76)	0.094	0.919	0.042
Current pain intensity, 0-10NRS	2.6 (2.4)	3.3 (2.6)	2.6 (2.7)	3.9 (2.9)	0.041	0.002	0.326
Pain walking on flat*	60 (28)	63 (37)	7 (25)	156 (55)	0.422	<0.001	0.007
Incident pain	144 (61)	126 (71)	27 (90)	205 (68)	0.145	0.047	0.777
Duration of morning stiffness							
≤30 mins	122 (52)	104 (58)	16 (53)	188 (63)			
>30 mins	16 (7)	8 (4)	2 (7)	16 (5)	0.199	0.130	0.952
Stiffness on waking*	68 (32)	74 (44)	6 (21)	168 (59)	0.045	<0.001	0.022
Inactivity gelling	16 (70)	141 (79)	21 (70)	259 (86)	0.092	0.002	0.098
Swollen in past month	67 (28)	50 (28)	8 (27)	131 (44)	0.498	0.001	0.017
Dramatic swelling ever	22 (9)	24 (14)	5 (17)	62 (21)	0.273	<0.001	0.041
Locking	27 (11)	20 (11)	2 (7)	40 (13)	0.887	0.521	0.308
Giving way	62 (26)	62 (35)	10 (33)	100 (33)	0.078	0.354	0.395
Significant interference with daily activities	47 (20)	48 (27)	3 (10)	101 (34)	0.250	0.088	0.683
Difficulty descending stairs*	57 (27)	74 (43)	6 (21)	166 (59)	0.019	<0.001	0.053
Intercondylar gap>0cm	42 (18)	32 (18)	3 (10)	84 (28)	0.504	0.005	<0.001
Intermalleolar gap>0cm	75 (32)	51 (29)	5 (17)	100 (33)	0.627	0.926	0.923
PFJ compression test							
Glide pain	44 (19)	35 (20)	6 (20)	65 (22)			
Compression pain	61 (26)	59 (33)	6 (20)	94 (31)	0.202	0.383	0.273
Knee effusion							
Mild	38 (16)	32 (18)	6 (20)	104 (35)			
Moderate/gross	11 (5)	17 (10)	3 (10)	56 (19)	0.393	<0.001	<0.001
Fixed flexion deformity	3 (1)	13 (7)	2 (7)	72 (24)	0.016	<0.001	<0.001
Bony enlargement							
Possible	63 (27)	52 (29)	13 (43)	94 (31)			
Definite	20 (9)	16 (9)	1 (3)	84 (28)	0.659	<0.001	<0.001
Mediolateral instability							
Possible	73 (31)	53 (30)	12 (40)	75 (25)			
Definite	49 (21)	34 (19)	5 (17)	102 (34)	0.980	0.055	0.019

Additional File 1. continued

	Pattern of knee OA				Binary logistic regression†		
	NONE	ISO-PF	ISO-TF	COMB	ISO-PF vs NONE	COMB vs NONE	COMB vs ISO-PF
Knee extensor strength, mmHg							
201-299	72 (31)	52 (29)	5 (17)	70 (23)			
141-200	67 (29)	34 (19)	9 (30)	83 (28)			
0-140	46 (20)	47 (26)	8 (27)	83 (28)	*0.141*	*0.156*	*0.375*
Knee flexor strength, mmHg							
141-184	70 (30)	36 (20)	8 (27)	70 (23)			
101-139	56 (24)	39 (22)	10 (33)	66 (22)			
0-100	61 (26)	45 (25)	6 (20)	90 (30)	*0.202*	*0.326*	*0.773*
Coarse crepitus							
Possible	33 (14)	30 (17)	4 (13)	70 (23)			
Definite	26 (11)	36 (20)	3 (10)	95 (32)	*0.019*	*<0.001*	*0.003*
Knee flexion ROM, degrees: mean (SD)	135.0 (10.7)	134.2 (11.3)	129.8 (14.1)	125.9 (14.2)	*0.666*	*<0.001*	*<0.001*
TF joint line tenderness	116 (49)	87 (49)	15 (50)	158 (52)	*0.838*	*0.870*	*0.850*
Multiple tender points	39 (17)	27 (15)	4 (13)	52 (17)	*0.947*	*0.569*	*0.568*
Single-leg standing balance, secs							
10-29	53 (27)	49 (28)	7 (23)	60 (20)			
4-9	63 (27)	50 (28)	6 (20)	84 (28)			
0-3	37 (16)	36 (20)	5 (17)	95 (20)	*0.394*	*0.430*	*0.371*

†from binary logistic regression, adjusted for age, gender, and body mass index; ‡ pain, aching or stiffness on most days in past month; * item from WOMAC LK3.0, dichotomised at moderate or worse; § item from Chronic Pain Grade
