# Supplementary material for: Clinical features of symptomatic patellofemoral joint osteoarthritis
Source: Arthritis Res Ther. 2012 Mar 14;14(2):R63. doi: 10.1186/ar3779 (PMC3446431; doi:10.1186/ar3779)
Supplement: Additional file 2 — Content and performance of multivariable models: 'any OA'. Full reporting, including posterior probability distributions, of the multivariable binary logistic regression models for each pairwise comparison of participants with no radiographic OA, isolated patellofemoral joint OA, or combined patellofemoral/tibiofemoral joint OA, using the less stringent cut-off of 'any OA'. [file ar3779-S2.PDF]

Additional File 2. Content and performance of multivariable models: ‘any OA’

(a) Isolated PFJOA vs No OA

	aOR (95%CI)
Age, years*	1.05 (1.02, 1.08)
Female gender	0.34 (0.21, 0.53)
BMI, kg/m^2*	1.05 (1.00, 1.10)
Difficulty descending stairs	1.83 (1.13, 2.96)
Crepitus (ref: none)	
Possible	1.20 (0.65, 2.22)
Definite	2.46 (1.32, 4.60)

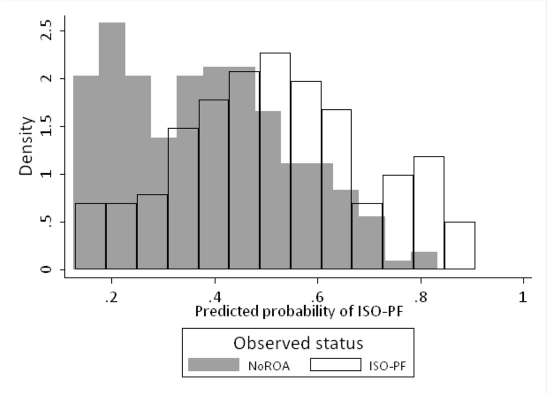

AUC =0.71 (0.66, 0.76)
GOF p = 0.70
Model with age, sex, BMI: AUC = 0.69 (0.64, 0.74)

(b) Combined PFJ/TFJOA vs No OA

	aOR (95%CI)
Age, years*	1.09 (1.05, 1.12)
Female gender	0.44 (0.26, 0.73)
BMI, kg/m^2*	1.08 (1.02, 1.14)
Onset following injury	2.18 (1.07, 4.44)
Whole leg pain	0.28 (0.13, 0.61)
Stiffness on waking	1.92 (1.10, 3.34)
Difficulty descending stairs	2.53 (1.40, 4.57)
Knee effusion (ref: none)	
Mild	3.08 (1.75, 5.42)
Moderate/gross	2.64 (1.12, 6.21)
Fixed flexion deformity	7.58 (2.08, 27.58)
Crepitus (ref: none)	
Possible	1.92 (1.09, 3.77)
Definite	3.38 (1.75, 6.55)
Knee flexion ROM, degrees*	0.96 (0.94, 0.99)

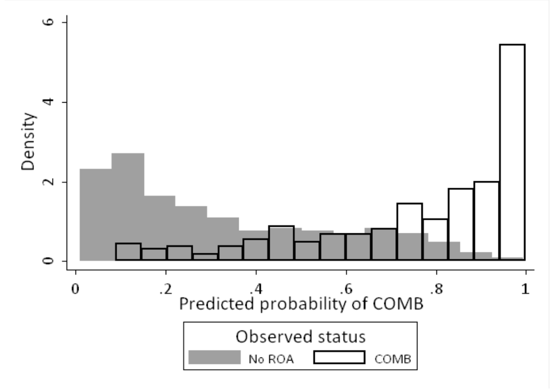

AUC = 0.88 (0.85, 0.91)
GOF p = 0.48
Model with age, sex, BMI: AUC = 0.77 (0.73, 0.81)

(c) Combined PFJ/TFJOA vs Isolated PFJOA

	aOR (95%CI)
Age, years*	1.05 (1.02, 1.08)
Female gender	1.70 (1.07, 2.69)
BMI, kg/m^2*	1.06 (1.01, 1.12)
Intercondylar gap > 0cm	2.11 (1.18, 3.75)
Knee effusion (ref: none)	
Mild	2.82 (1.70, 4.69)
Moderate/gross	1.56 (0.79, 3.08)
Bony enlargement (ref: none)	
Possible	1.25 (0.77, 2.03)
Definite	3.01 (1.56, 5.81)
Fixed flexion deformity	2.11 (1.04, 4.28)
Knee flexion ROM, degrees*	0.96 (0.94, 0.99)

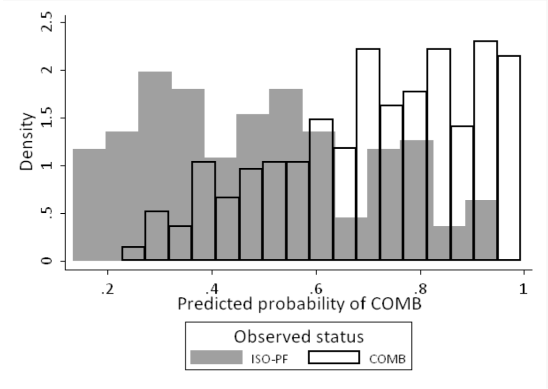

AUC = 0.78 (0.73, 0.82)
GOF p = 0.21
Model with age, sex, BMI: AUC = 0.66 (0.61, 0.71)

aOR adjusted odds ratio from binary logistic regression; 95%CI 95 percent confidence interval; AUC area under the ROC curve; GOF Hosmer-Lemeshow goodness of fit statistic; * aOR per 1-unit increase in the indicator
