# Supplementary material for: Clinical features of symptomatic patellofemoral joint osteoarthritis
Source: Arthritis Res Ther. 2012 Mar 14;14(2):R63. doi: 10.1186/ar3779 (PMC3446431; doi:10.1186/ar3779)
Supplement: Additional file 3 — Descriptive characteristics and univariable analysis: 'moderate to severe OA'. This file provides the full descriptive characteristics and pairwise univariable comparison of participants with (1) no radiographic OA, (2) isolated patellofemoral joint OA, isolated tibiofemoral joint OA, or combined patellofemoral/tibiofemoral joint OA, using the more stringent cut-off of 'moderate to severe OA'. [file ar3779-S3.PDF]

Additional file 3. Descriptive characteristics and univariable analysis: ‘moderate-severe OA’

	Pattern of knee OA				Binary logistic regression†					
	NONE/MILD	ISO-PF	ISO-TF	COMB	ISO-PF vs No-mild	ISO-TF vs No-mild	COMB vs No-mild	ISO-TF vs ISO-PF	COMB vs ISO-PF	COMB vs ISO-TF
	n=453	n=99	n=123	n=70	*p*	*p*	*P*	*P*	*p*	*p*
Age, years: mean (SD)	62.7 (7.1)	69.4 (9.0)	69.1 (8.3)	68.9 (7.8)	*<0.001*	*<0.001*	*<0.001*	*0.645*	*0.581*	*0.866*
Female gender	255 (56)	63 (64)	53 (43)	36 (51)	*0.135*	*0.018*	*0.564*	*0.002*	*0.103*	*0.270*
BMI, kg/m^2 : mean (SD)	28.9 (4.9)	30.2 (4.9)	30.6 (5.4)	32.0 (6.4)	*<0.001*	*<0.001*	*<0.001*	*0.462*	*0.028*	*0.152*
1st degree relative with arthritis	290 (64)	51 (52)	70 (57)	43 (61)	*0.168*	*0.987*	*0.973*	*0.223*	*0.239*	*0.810*
Clinical hand OA	78 (17)	21 (21)	21 (17)	12 (17)	*0.858*	*0.270*	*0.570*	*0.955*	*0.762*	*0.752*
Previous menisectomy	7 (2)	1 (1)	13 (11)	5 (7)	-	-	-	-	-	-
Contralateral TKR	2 (<1)	4 (4)	4 (3)	2 (3)	-	-	-	-	-	-
Time since onset										
1-5 yrs	163 (36)	34 (34)	37 (31)	23 (33)						
5-10 yrs	91 (20)	19 (19)	25 (20)	15 (21)						
>10 yrs	126 (28)	40 (40)	55 (45)	26 (37)	*0.003*	*0.001*	*0.086*	*0.995*	*0.928*	*0.754*
Gradual onset	305 (67)	67 (68)	92 (75)	53 (76)	*0.816*	*0.164*	*0.276*	*0.390*	*0.502*	*0.867*
Onset following injury	57 (13)	9 (9)	24 (20)	17 (24)	*0.462*	*0.067*	*0.011*	*0.026*	*0.006*	*0.459*
Whole leg pain	59 (13)	9 (9)	12 (10)	8 (11)	*0.227*	*0.368*	*0.647*	*0.674*	*0.902*	*0.918*
Pain days in past 6mo										
31-89	114 (25)	21 (21)	32 (26)	25 (36)						
90+	92 (20)	37 (37)	47 (38)	25 (36)	*0.020*	*0.007*	*0.008*	*0.504*	*0.128*	*0.491*
Frequent symptoms‡	171 (38)	54 (55)	73 (59)	42 (60)	*0.013*	*0.001*	*0.007*	*0.543*	*0.664*	*0.916*
Bilateral knee pain	330 (73)	83 (86)	89 (72)	57 (81)	*0.043*	*0.389*	*0.475*	*0.022*	*0.452*	*0.218*
Current pain intensity, 0-10NRS§	2.7 (2.5)	4.2 (2.8)	4.0 (2.9)	4.7 (2.9)	*<0.001*	*0.007*	*<0.001*	*0.363*	*0.486*	*0.184*
Pain walking on flat*	129 (31)	41 (44)	72 (62)	44 (65)	*0.152*	*<0.001*	*<0.001*	*0.021*	*0.021*	*0.756*
Incident pain	299 (66)	70 (71)	86 (70)	47 (67)	*0.276*	*0.770*	*0.944*	*0.547*	*0.522*	*0.754*
Duration of morning stiffness										
≤30 mins	244 (54)	63 (64)	76 (62)	47 (67)						
>30 mins	25 (6)	6 (6)	7 (6)	4 (6)	*0.469*	*0.375*	*0.168*	*0.942*	*0.630*	*0.890*
Stiffness on waking*	153 (36)	47 (52)	70 (60)	46 (67)	*0.123*	*<0.001*	*0.001*	*0.308*	*0.136*	*0.422*
Inactivity gelling	337 (74)	83 (84)	104 (85)	63 (90)	*0.191*	*0.118*	*0.021*	*0.933*	*0.152*	*0.326*
Swollen in past month	115 (25)	46 (47)	56 (46)	39 (56)	*0.002*	*<0.001*	*<0.001*	*0.568*	*0.121*	*0.217*
Dramatic swelling ever	49 (11)	26 (26)	16 (13)	22 (31)	*<0.001*	*0.413*	*<0.001*	*0.007*	*0.513*	*0.001*
Locking	48 (11)	9 (9)	18 (15)	14 (20)	*0.777*	*0.283*	*0.031*	*0.132*	*0.027*	*0.425*
Giving way	128 (28)	41 (41)	37 (30)	28 (40)	*0.052*	*0.757*	*0.254*	*0.083*	*0.882*	*0.242*
Significant interference with daily activities§	92 (20)	34 (34)	43 (35)	30 (43)	*0.048*	*0.037*	*0.005*	*0.875*	*0.435*	*0.509*
Difficulty descending stairs	132 (31)	54 (58)	71 (61)	46 (68)	*0.001*	*<0.001*	*<0.001*	*0.494*	*0.266*	*0.619*
Intercondylar gap>0cm	88 (20)	11 (11)	48 (39)	14 (20)	*0.444*	*<0.001*	*0.235*	*<0.001*	*0.066*	*0.024*

Additional file 3. continued

	Pattern of knee OA				Binary logistic regression†					
	NONE/MILD	ISO-PF	ISO-TF	COMB	ISO-PF vs No-mild	ISO-TF vs No-mild	COMB vs No-mild	ISO-TF vs ISO-PF	COMB vs ISO-PF	COMB vs ISO-TF
	n=453	n=99	n=123	n=70	*P*	*p*	*P*	*p*	*p*	*p*
Intermalleolar gap>0cm	130 (29)	46 (47)	29 (24)	26 (37)	*0.009*	*0.335*	*0.285*	*0.001*	*0.161*	*0.137*
PFJ compression test										
Glide pain	88 (20)	25 (25)	23 (19)	14 (20)						
Compression pain	115 (25)	44 (44)	41 (33)	20 (29)	*0.001*	*0.371*	*0.985*	*0.065*	*0.020*	*0.419*
Knee effusion										
Mild	85 (19)	31 (31)	42 (34)	22 (31)						
Moderate/gross	23 (5)	19 (19)	25 (20)	20 (29)	*<0.001*	*<0.001*	*<0.001*	*0.803*	*0.199*	*0.427*
Fixed flexion deformity	21 (5)	7 (7)	35 (29)	27 (39)	*0.533*	*<0.001*	*<0.001*	*<0.001*	*<0.001*	*0.114*
Bony enlargement										
Possible	126 (28)	30 (31)	45 (37)	21 (30)						
Definite	41 (9)	16 (17)	35 (29)	29 (41)	*0.279*	*<0.001*	*<0.001*	*0.013*	*<0.001*	*0.074*
Mediolateral instability										
Possible	139 (31)	23 (24)	39 (32)	12 (17)						
Definite	93 (21)	23 (24)	39 (32)	35 (50)	*0.231*	*0.105*	*<0.001*	*0.007*	*<0.001*	*0.014*
Knee extensor strength, mmHg										
201-299	140 (31)	16 (16)	31 (26)	12 (17)						
141-200	111 (25)	26 (26)	34 (28)	22 (31)						
0-140	90 (20)	46 (47)	27 (22)	21 (30)	*0.001*	*0.358*	*0.030*	*0.011*	*0.301*	*0.578*
Knee flexor strength, mmHg										
141-184	120 (27)	16 (16)	31 (26)	17 (24)						
101-139	99 (22)	30 (30)	23 (19)	19 (27)						
0-100	110 (24)	39 (39)	34 (28)	19 (27)	*0.071*	*0.732*	*0.883*	*0.166*	*0.559*	*0.659*
Coarse crepitus										
Possible	74 (14)	20 (20)	28 (23)	15 (21)						
Definite	60 (13)	33 (33)	39 (32)	28 (40)	*<0.001*	*<0.001*	*<0.001*	*0.868*	*0.603*	*0.464*
Knee flexion ROM, degrees:										
mean (SD)	134.4 (11.1)	130.0 (12.5)	124.8 (13.9)	120.3 (15.9)	*0.355*	*<0.001*	*<0.001*	*0.001*	*<0.001*	*0.090*
TF joint line tenderness	216 (48)	58 (59)	66 (54)	36 (51)	*0.561*	*0.625*	*0.487*	*0.956*	*0.463*	*0.423*
Multiple tender points	67 (15)	26 (26)	16 (13)	46 (19)	*0.098*	*0.374*	*0.968*	*0.025*	*0.158*	*0.559*
Single-leg standing balance, secs										
10-29	117 (26)	19 (19)	23 (19)	10 (15)						
4-9	120 (27)	29 (30)	34 (28)	20 (29)						
0-3	69 (15)	35 (36)	42 (34)	27 (39)	*0.117*	*0.139*	*0.049*	*0.965*	*0.905*	*0.878*

†from binary logistic regression, adjusted for age, gender, and body mass index; ‡ pain, aching or stiffness on most days in past month; * item from WOMAC LK3.0, dichotomised at moderate or worse; § item from Chronic Pain Grade
