# Supplementary material for: Clinical features of symptomatic patellofemoral joint osteoarthritis
Source: Arthritis Res Ther. 2012 Mar 14;14(2):R63. doi: 10.1186/ar3779 (PMC3446431; doi:10.1186/ar3779)
Supplement: Additional file 4 — Content and performance of multivariable models: 'moderate to severe OA'. Full reporting, including posterior probability distributions, of the multivariable binary logistic regression models for each pairwise comparison of participants with no radiographic OA, isolated patellofemoral joint OA, isolated tibiofemoral joint OA, or combined patellofemoral/tibiofemoral joint OA, using the more stringent cut-off of 'moderate to severe OA'. [file ar3779-S4.PDF]

Additional File 4. Content and performance of multivariable models: ‘moderate-severe OA’

(a) Isolated PFJOA vs No/mild OA

	aOR (95%CI)
Age, years*	1.12 (1.08, 1.16)
Female gender	0.78 (0.42, 1.46)
BMI, kg/m^2*	1.08 (1.02, 1.14)
Time since onset (ref: <1 yr)	
1-5 yrs	4.88 (1.65, 14.42)
5-10 yrs	4.91 (1.57, 15.36)
>10 yrs	4.51 (1.53, 13.67)
Swollen in past month	1.68 (0.95, 2.98)
Intermalleolar gap > 0cm	2.26 (1.28, 3.98)
Knee effusion (ref: none)	
Mild	2.54 (1.40, 4.60)
Moderate/gross	3.89 (1.68, 8.99)
Knee extensor strength (ref: 300+ mmHg)	
201-299	0.87 (0.34, 2.19)
141-200	1.63 (0.64, 4.15)
0-140	2.98 (1.20, 7.39)
Crepitus (ref: none)	
Possible	1.58 (0.79, 3.15)
Definite	4.26 (2.18, 8.32)

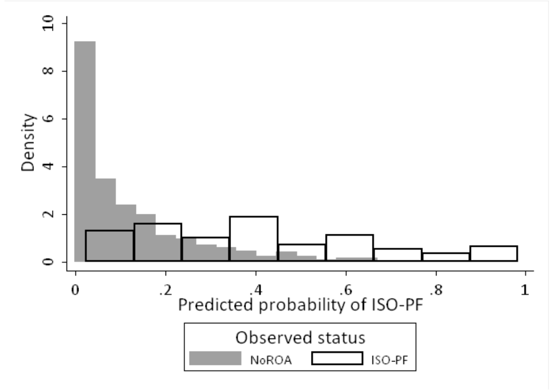

GOF p = 0.80
AUC = 0.85 (0.82, 0.89)
Model with age, sex, BMI: AUC = 0.75 (0.70, 0.80)

(b) Isolated TFJOA vs No/mild OA

	aOR (95%CI)
Age, years*	1.10 (1.06, 1.13)
Female gender	0.85 (0.46, 1.55)
BMI, kg/m^2*	1.08 (1.02, 1.14)
Time since onset (ref: <1 yr)	
1-5 yrs	4.14 (1.36, 12.56)
5-10 yrs	3.26 (1.02, 10.41)
>10 yrs	5.19 (1.73, 15.57)
Swollen in past month	2.03 (1.16, 3.58)
Difficulty descending stairs	1.89 (1.10, 3.25)
Intercondylar gap > 0cm	3.27 (1.71, 6.27)
Knee effusion (ref: none)	
Mild	2.32 (1.30, 4.14)
Moderate/gross	3.41 (1.50, 7.78)
Fixed flexion deformity	3.25 (1.50, 7.03)
Knee flexion ROM, degrees*	0.97 (0.94, 0.99)

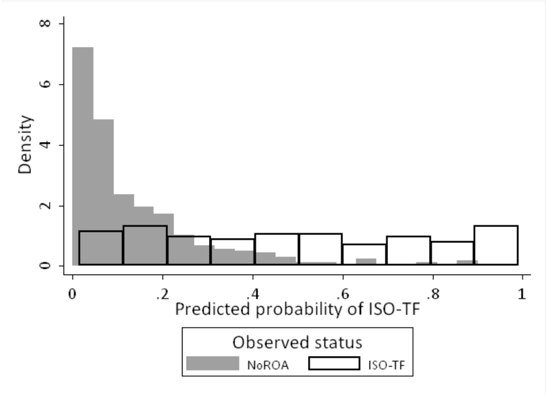

GOF p = 0.68
AUC = 0.86 (0.82, 0.90)
Model with age, sex, BMI: AUC = 0.75 (0.70, 0.80)

(c) Combined PFJ/TFJOA vs No/mild OA

	aOR (95%CI)
Age, years*	1.10 (1.05, 1.15)
Female gender	0.71 (0.32, 1.56)
BMI, kg/m^2*	1.12 (1.05, 1.20)
Onset following injury	2.53 (1.06, 6.04)
Swollen in past month	2.34 (1.09, 5.03)
Difficulty descending stairs	2.35 (1.11, 5.01)
Knee effusion (ref: none)	
Mild	2.64 (1.18, 5.95)
Moderate/gross	6.08 (2.29, 16.16)
Fixed flexion deformity	9.16 (3.58, 23.39)
Mediolateral instability	
Possible	0.71 (0.28, 1.77)
Definite	2.41 (1.05, 5.49)
Knee flexion ROM, degrees*	0.97 (0.95, 1.00)

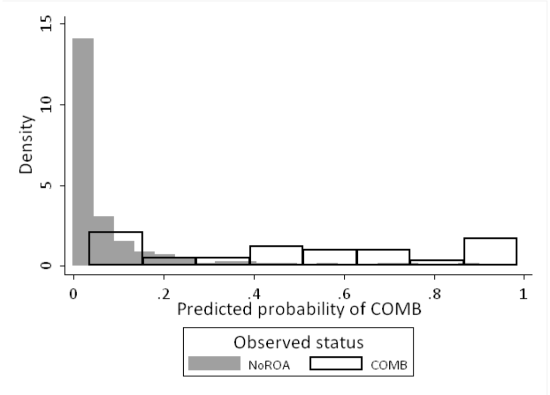

GOF p = 0.77
AUC = 0.92 (0.89, 0.95)
Model with age, sex, BMI: AUC = 0.78 (0.72, 0.84)

aOR adjusted odds ratio from binary logistic regression; AUC area under the ROC curve; GOF Hosmer-Lemeshow goodness of fit statistic; * aOR per 1-unit increase in the indicator

Additional File 4. continued

(d) Isolated TFJOA vs Isolated PFJOA

	aOR (95%CI)
Age, years	0.99 (0.95, 1.03)
Female gender	0.77 (0.34, 1.76)
BMI, kg/m^2	0.99 (0.91, 1.08)
Onset following injury	5.24 (1.79, 15.4)
Bilateral knee pain	0.30 (0.12, 0.76)
Dramatic swelling ever	0.31 (0.12, 0.75)
Intercondylar gap>0cm	3.81 (1.44, 10.2)
Intermalleolar gap>0cm	0.40 (0.18, 0.86)
Bony enlargement (ref: none)	
Possible	2.23 (1.01, 4.89)
Definite	4.06 (1.52, 10.8)
Knee extensor strength (ref: 300+ mmHg)	
201-299	0.52 (0.16, 1.70)
141-200	0.70 (0.22, 2.24)
0-140	0.22 (0.06, 0.72)
Knee flexion ROM, degrees	0.92 (0.89, 0.96)

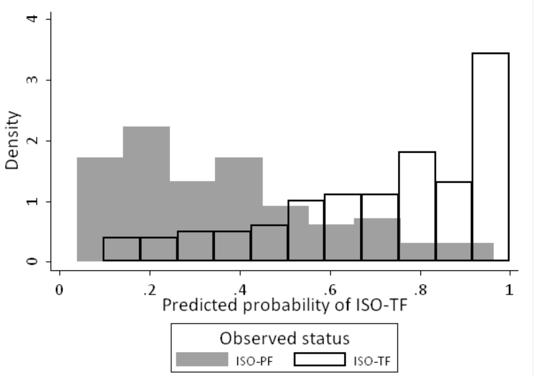

GOF p = 0.37
AUC = 0.85 (0.79, 0.90)
Model with age, sex, BMI: AUC = 0.61 (0.53, 0.69)

(e) Combined PFJ/TFJOA vs Isolated PFJOA

	aOR (95%CI)
Age, years	1.01 (0.96, 1.07)
Female gender	0.93 (0.40, 2.19)
BMI, kg/m^2	1.06 (0.98, 1.15)
Onset following injury	3.80 (1.33, 10.9)
Locking	4.28 (1.31, 14.0)
Bony enlargement (ref: none)	
Possible	3.31 (1.24, 8.81)
Definite	5.21 (1.92, 14.2)
Fixed flexion deformity	5.40 (1.80, 16.2)
PFJ compression (ref: none)	
Glide pain	0.41 (0.15, 1.14)
Compression pain	0.18 (0.06, 0.52)
Knee flexion ROM, degrees	0.95 (0.92, 0.99)

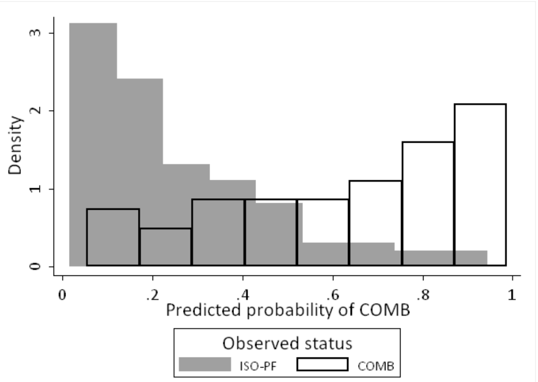

GOF p = 0.62
AUC = 0.85 (0.79, 0.91)
Model with age, sex, BMI: AUC = 0.62 (0.53, 0.71)

(f) Combined PFJ/TFJOA vs Isolated TFJOA

	aOR (95%CI)
Age, years	1.00 (0.96, 1.04)
Female gender	0.84 (0.41, 1.76)
BMI, kg/m^2	1.03 (0.97, 1.10)
Dramatic swelling ever	2.58 (1.17, 5.70)
Intercondylar gap>0cm	0.39 (0.16, 0.92)
Mediolateral instability (ref: none)	
Possible	0.61 (0.25, 1.48)
Definite	1.92 (0.89, 4.13)

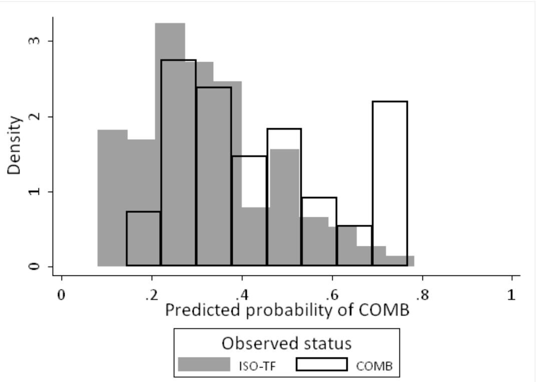

GOF p = 0.64
AUC = 0.70 (0.62, 0.77)
Model with age, sex, BMI: AUC = 0.57 (0.48, 0.66)

aOR adjusted odds ratio from binary logistic regression; AUC area under the ROC curve; GOF Hosmer-Lemeshow goodness of fit statistic; * aOR per 1-unit increase in the indicator
