# Supplementary material for: Clinical features of symptomatic patellofemoral joint osteoarthritis
Source: Arthritis Res Ther. 2012 Mar 14;14(2):R63. doi: 10.1186/ar3779 (PMC3446431; doi:10.1186/ar3779)
Supplement: Additional file 5 — Sensitivity analysis of the association between varus/valgus malalignment and pattern of joint involvement: 'moderate to severe OA'. Demonstration of the strengthening of association between malalignment and pattern of joint involvement when using compartment-specific definitions. [file ar3779-S5.PDF]

Additional File 5. Sensitivity analysis of the association between varus/valgus malalignment and pattern of joint involvement: 'moderate-severe OA'

(a) Broad case definition
In the table below, isolated tibiofemoral joint OA and isolated patellofemoral joint OA could be any mixture of medial and lateral compartment disease

	ISO-TF* N=123	ISO-PF* N=99	ISO-TF vs ISO-PF aOR (95%CI)†
Intercondylar gap>0cm	48 (39)	11 (11)	4.97 (2.23, 11.07)
Intermalleolar gap>0cm	29 (24)	46 (46)	0.36 (0.20, 0.67)

* Defined as per Table 1
† Odds ratio and 95% confidence interval adjusted for age, gender, BMI

(b) Narrow case definition
In the table below, isolated tibiofemoral joint OA has been restricted to medial compartment disease and isolated patellofemoral joint OA has been restricted to lateral compartment disease

	Medial ISO-TF‡ N=85	Lateral ISO-PF§ N=50	ISO-TF vs ISO-PF aOR (95%CI)†
Intercondylar gap>0cm	43 (51)	5 (10)	9.96 (3.31, 29.96)
Intermalleolar gap>0cm	12 (14)	24 (48)	0.17 (0.07, 0.41)

‡ Defined as isolated tibiofemoral joint 'moderate-severe OA' with medial TFJ compartment joint space narrowing grade 2-3 and lateral TFJ joint space narrowing grade 0-1 (PA view)
§ Defined as isolated patellofemoral joint 'moderate-severe OA' with lateral PFJ compartment joint space narrowing grade 2-3 and medial PFJ joint space narrowing grade 0-1 (skyline view)
† Odds ratio and 95% confidence interval adjusted for age, gender, BMI

In each case the strength of association is effectively doubled by using a narrower compartment-specific definition, underscoring the strong association between varus malalignment (estimated using intercondylar gap in standing) and medial tibiofemoral joint OA and between valgus malalignment (intermalleolar gap in standing) and lateral patellofemoral joint OA.
